# Pan-cancer transcriptomic analysis of CNS tumor stroma identifies a population of perivascular fibroblasts that predict poor immunotherapy response in glioblastoma patients

**DOI:** 10.21203/rs.3.rs-2931886/v1

**Published:** 2023-05-15

**Authors:** Maksym Zarodniuk, Alexander Steele, Xin Lu, Jun Li, Meenal Datta

**Affiliations:** 1 Department of Aerospace and Mechanical Engineering, University of Notre Dame; 2 Department of Electrical Engineering, University of Notre Dame; 3 Department of Biological Sciences, University of Notre Dame; 4 Department of Applied and Computational Mathematics and Statistics, University of Notre Dame

## Abstract

Excessive deposition of extracellular matrix (ECM) is a hallmark of solid tumors; however, it remains poorly understood which cellular and molecular components contribute to the formation of ECM stroma in central nervous system (CNS) tumors. Here, we undertook a pan-CNS analysis of retrospective gene expression datasets to characterize inter- and intra-tumoral heterogeneity of ECM remodeling signatures in both adult and pediatric CNS disease. We found that CNS lesions – glioblastoma in particular – can be divided into two ECM-based subtypes (ECM^hi^ and ECM^lo^) that are influenced by the presence of perivascular cells resembling cancer-associated fibroblasts (CAFs). We show that perivascular fibroblasts activate chemoattractant signaling pathways to recruit tumor-associated macrophages, and promote an immune-evasive, stem-like cancer cell phenotype. Our analysis reveals that perivascular fibroblasts are correlated with unfavorable response to immune checkpoint blockade in glioblastoma and poor patient survival across a subset of CNS tumors. We provide insights into novel stroma-driven mechanisms underlying immune evasion and immunotherapy resistance in CNS tumors like glioblastoma, and discuss how targeting these perivascular fibroblasts may prove an effective approach to improving treatment response and patient survival in a variety of CNS tumors.

## Introduction

CNS tumors comprise a highly heterogeneous group of malignancies that originate from different cell types and affect various anatomical structures. Despite recent advances in immunotherapeutic approaches to treat solid tumors, survival for many types of CNS cancers has not improved in the past 10 years (Jones et al. 2019; Lapointe, Perry, and Butowski 2018). Thus, there is an urgent need for a better understanding of the underlying mechanisms governing disease progression and treatment resistance.

Tumor stroma, composed of extracellular matrix (ECM) and specialized connective tissue cells that include fibroblasts and mesenchymal stromal cells, has been shown to shape antitumor immunity and response to immunotherapy across a broad range of epithelial tumors such as breast, colon, and pancreatic carcinomas (Turley, Cremasco, and Astarita 2015; Loeffler et al. 2006; Liao et al. 2009; Özdemir et al. 2014; Rhim et al. 2014). However, little is known about the immunomodulatory roles of the brain tumor stroma, and the distinct cellular makeup of the brain makes it challenging to extrapolate findings from cancers arising in peripheral organs.

Parenchyma of the CNS is often regarded as “fibro-privileged” since fibrogenic cells are absent in the normal neural parenchyma and are instead restricted to perivascular and meningeal niches (Fernández-Klett and Priller 2014; Yang et al. 2022). This is in line with the lack of fibrillar collagens in the brain tumor parenchyma (Mohiuddin and Wakimoto 2021). It has been shown in a number of neuropathologic conditions such as traumatic brain injury, ischemic stroke, and multiple sclerosis that perivascular stromal cells can detach from the vasculature, migrate into the parenchyma, and form a dense fibrotic scar (David O. Dias et al. 2021), thereby inhibiting axonal regeneration (David Oliveira Dias et al. 2018). However, the exact contribution of perivascular stromal cells to the brain tumor ECM remains unclear. This is partly due to the limited evidence supporting the existence of fibroblasts in primary brain tumors that are otherwise commonly found in the ECM-rich stroma of highly desmoplastic extracranial tumors such as breast, prostate, and pancreatic carcinomas (Kalluri and Zeisberg 2006; Jain et al. 2021; Galbo et al. 2022). Both extracranial cancer-associated fibroblasts (CAFs) and CAF-derived ECM have been linked to suppression of antitumor immune responses (Kraman et al. 2010; Erez et al. 2010; Pickup, Mouw, and Weaver 2014); however, evidence supporting their immunosuppressive roles in brain tumors is lacking. Therefore, a better understanding of the cellular origin and role of stroma in the brain tumor microenvironment (TME) can reveal novel mechanisms of immune escape and guide the design of new strategies to improve immunotherapeutic outcomes in brain tumors.

To this end, we performed a meta-analysis of publicly available gene expression datasets from multiple cohorts of pediatric and adult primary CNS tumors. We focused our attention on “core matrisome” – the ensemble of genes encoding structural components of the ECM, such as collagens and proteoglycans (Naba et al. 2016), the latter of which constitute a major component of brain tumor ECM (Wade et al. 2013; Mohiuddin and Wakimoto 2021). Through unsupervised analysis of gene expression data, we identified a subset of glioblastoma (GBM) tumors characterized by overexpression of transcripts of extracellular matrix (ECM) components and the presence of perivascular stromal cells resembling cancer-associated fibroblasts (CAF-like cells). This signature was conserved across a broad range of CNS malignancies and predicted poor patient survival in a subset of tumors. Interrogation of retrospective data from recent anti-PD1 immunotherapy trials (Cloughesy et al. 2019; J. Zhao et al. 2019) revealed an association between the presence of perivascular fibroblasts and immunotherapy resistance. In this study, we propose several mechanisms by which perivascular fibroblasts can contribute to immune evasion and immunotherapy resistance in CNS disease.

## Results

### Unsupervised analysis of extracellular matrix expression in glioblastoma reveals two states that are conserved in other CNS tumors

**Figure 1.**
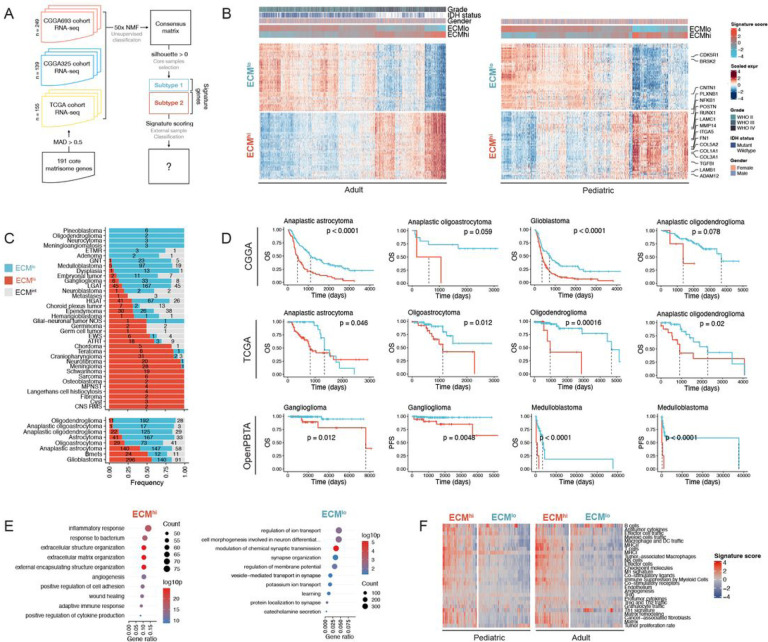
Unsupervised analysis of retrospective RNA-seq data reveals two extracellular matrix states in glioblastoma that are prognostic of clinical outcome and conserved across multiple central nervous system cancers. **(A)** Outline of the computational workflow. **(B)** ECM^hi^ and ECM^lo^ signature gene expression in adult (left) and pediatric (right) central nervous system tumors. Select signature genes are labeled. **(C)** Frequency of ECM states across different CNS tumors. **(D)** Kaplan-Meier survival curves for ECM^hi^ (red) and ECM^lo^ (blue) tumors of different histologies. Only tumors with a statistically significant effect (p<0.05, log-rank test) are shown. **(E)** Gene ontology (GO) terms enriched in ECM^hi^ (left) and ECM^lo^ (right) states in adult gliomas. Top 10 GO terms are shown. **(F)** Functional gene expression signatures from Bagaev et al. (2021) in pediatric and adult brain tumors, grouped by ECM state.

Glioblastoma (GBM) tissue, unlike that of low-grade gliomas (LGGs), has been shown to display extensive and heterogeneous ECM deposition and tissue remodeling (Dinevska et al. 2022). Thus, we began our analysis with an unbiased characterization of the ECM transcriptome in GBM (Figure 1A). First, we batch corrected (Figure S1A) publicly available GBM RNA-sequencing (RNA-seq) profiles from three independent datasets (TCGA, CGGA-693, CGGA-325) across 558 patients and performed non-negative matrix factorization (NMF) on the core matrisome gene expression matrix, thereby limiting our analysis to genes encoding structural components of ECM (Naba et al. 2016). Based on cophenetic correlation coefficient (Figure S1E) and results from independent hierarchical clustering (Figures S1C–D), we grouped the data into two classes and defined a set of 505 samples as core samples based on a positive silhouette coefficient (Figures S1B).

In order to characterize the two classes/subtypes and classify external samples, we performed differential expression analysis on core samples between classes and, for each class, identified 49 signature genes as an intersection of top marker genes across all three datasets (Figure 1B, Supplementary Data). One cluster was characterized by overexpression of genes encoding ECM proteins, including collagens (*COL5A2*, *COL1A2*, *COL3A1*, *COL1A1*, *COL6A3*, *COL5A1*, *COL6A2*, *COL8A1*), glycoproteins (*LAMB1*, *LAMC1*, *SRPX2*, *POSTN*, *LTBP2*, *TGFBI*, *FN1*, *THBS1*), and matricellular proteins (*SERPINH1*, *PLOD1*,*MMP14*, *ADAM12*, *SERPINE1*, *LOXL2*). The clusters were defined as ECM^hi^, extracellular matrix high, (48.2%) and ECM^lo^, extracellular matrix low, (51.8%) to reflect their distinct ECM composition.

Next, to verify the existence of ECM^hi^ and ECM^lo^ subtypes in other CNS malignancies, we scored additional RNA-seq data spanning adult low-grade (n=1164) and pediatric (n=977) brain tumors as well as brain metastases (Bmets, n=47) for expression of ECM^hi^ and ECM^lo^ signature genes. We found 83% of adult gliomas, 80% of pediatric gliomas, and 77% of Bmets could be reliably classified into either subtype, indicating that these programs are conserved across a range of CNS malignancies beyond GBM. The remaining samples were defined by contemporaneous up- or down-regulation of ECM^hi^ and ECM^lo^ signature genes and could not be reliably classified. Principal component analysis (PCA) on core matrisome expression placed these samples between ECM^hi^ and ECM^low^ samples, suggesting that they display an intermediate ECM state (ECM^int^) (Figure S1F). In adult gliomas, ECM^hi^ subtype was associated with higher tumor grade, absence of IDH mutations, and mesenchymal subtype in GBM (Figure S1H) (P < 2.2E-16, Chi-Square Test). Pediatric tumors exhibited a highly skewed distribution of ECM states, where a number of tumors including craniopharyngioma (86%), neurofibroma (95%), meningioma (97%), and schwannoma (100%) were classified almost exclusively as ECM^hi^, whereas glioneuronal tumors (GNT, 3%) and medulloblastomas (4%) were classified almost exclusively as ECM^low^ (Figure 1C). However, we did not detect an association between ECM^hi^ score and the level of malignancy of pediatric brain tumors (Figure S1G), suggesting that the effect of fibrotic stroma may be context-specific. In astrocytoma, glioblastoma and mixed glioma tumors as well as pediatric ganglioglioma and medulloblastoma, we found an association between ECM^hi^ subtype and decreased patient survival (Figure 1D), suggesting that fibrosis can be prognostic of clinical outcome in a subset of CNS tumors.

Gene ontology (GO) enrichment analysis revealed an upregulation of inflammation, angiogenesis, and wound-healing response pathways in adult glioma ECM^hi^ tumors, whereas ECM^low^ samples were characterized by upregulation of neurosynaptic pathways (Figure 1E), suggesting an enrichment of non-neoplastic cells. Additionally, ECM^hi^ tumors upregulated numerous immune and stromal-related signatures (Figure 1F). Using data from the Ivy Glioblastoma Atlas Project (Ivy-GAP), we found that ECM^low^ signature was preferentially expressed at the leading edge (LE) and infiltrative region (IT) of the tumor, which are known to consist largely of non-neoplastic cells (Puchalski et al. 2018), whereas the ECM^hi^ signature was upregulated in regions corresponding to microvascular proliferation (CTmvp) and hyperplastic blood vessels (CThbv), in line with a recent study by (Hoogstrate et al. 2023) (Figure S1I). We also examined in situ hybridization (ISH) and adjacent hematoxylin and eosin (H&E) tissue sections annotated for the same histologic features. We found that ECM^hi^ hallmark genes *COL1A1*, *COL4A1* were expressed in CTmvp regions, suggesting that ECM^hi^ signature is spatially associated with GBM microvasculature (Figure S1J).

### ECM^hi^ tumors are characterized by the presence of perivascular fibroblasts whose enrichment predicts poor response to immunotherapy

**Figure 2.**
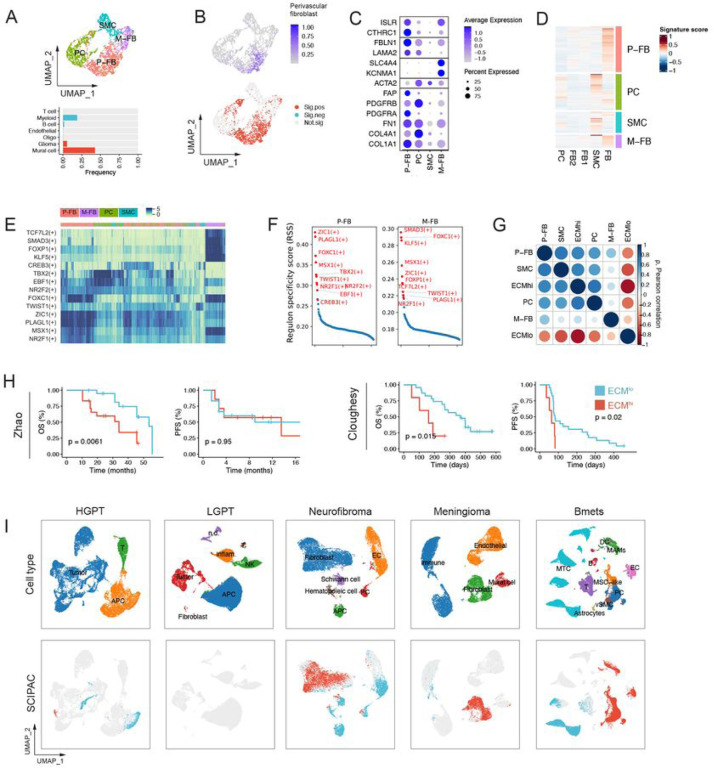
Cancer associated fibroblast-like cells of perivascular origin are present in ECMhi GBM and predict poor response to immunotherapy. **(A)** Top: a UMAP plot showing subpopulations of mural cells. Bottom: barplot showing the frequency of ECM^hi^-associated cells within each cell type. **(B)** UMAP embedding of mural cells from, colored by the enrichment of the perivascular fibroblast metagene (top) and SCIPAC predictions (bottom). **(C)** Marker genes identifying subpopulations of mural cells in GBM. P-FB – perivascular fibroblast; PC – pericyte; SMC – smooth muscle cell; M-FB – meningeal fibroblast. **(D)** Signature gene scores of murine perivascular cell types from Vanlandewijck et al. (2018). FB – fibroblast-like cell; SMC – smooth muscle cell; PC – pericyte. **(E)** SCENIC-inferred regulon activity in subpopulations of mural cells. Shown in red are top 10 regulons for each mural cell subpopulation based on regulon specificity score (RSS). **(F)** RSS of transcription factors in perivascular and meningeal fibroblasts; 10 transcription factors with the highest RSS are labeled in red. **(G)** Pearson correlation between ECM^hi^/ECM^lo^ metagenes and mural cell subpopulations. **(H)** Kaplan–Meier plot of overall and progression free survival for patients with high (red) or low (blue) presence of perivascular fibroblasts, treated with anti-PD1 therapy (P values were calculated using log-rank test). **(I)** UMAP embeddings of scRNA-seq data from different tumors, colored by cell type (top) and positive (red) or negative (blue) association with ECM^hi^ state, as predicted by SCIPAC. HGPT – high-grade pediatric tumors; LGPT – low-grade pediatric tumors.

The vascular microenvironment is an important brain tumor niche with a heterogeneous and not fully revealed cellular makeup (Charles and Holland 2010). In order to identify vascular/perivascular cellular components contributing to the ECM stroma in CNS tumors, we applied SCIPAC – a tool designed to identify phenotype-associated cells in single-cell RNA-sequencing (scRNA-seq) data – to a scRNA–seq dataset from 16 GBM patients (Figure S2A–C, S2E) (Abdelfattah et al. 2022). SCIPAC predicted 43% of PDGFRB+ ACTA2+ mural cells to be associated with the ECM^hi^ signature (Figure 2B). We sub-clustered and annotated mural cells based on heterogeneous gene expression signatures characterizing previously identified perivascular cell types in the human brain (Yang et al. 2022). We identified two clusters as perivascular fibroblasts (P-FB; *FBLN1*, *LAMA2*) and meningeal fibroblasts (M-FB; *SLC4A4*, *KCNMA1*), as well as separate clusters of pericytes (PC; *PDGFRB*, *COL4A1*), and smooth muscle cells (SMC; *ACTA2*) (Figure 2D). We found that 86% of P-FB cells were associated with the ECM^hi^ phenotype, suggesting their pro-fibrotic role in the GBM TME (Figure 2C). Additionally, we found that PV-FB cells resembled previously identified murine brain fibroblast-like cells (Vanlandewijck et al. 2018) (Figure 2D). To determine the contribution of these perivascular cells to ECM^hi^ and ECM^low^ states, we deconvoluted bulk gene expression profiles using a set of signature genes uniquely identifying each perivascular cell type. We found that ECM^hi^ metagene was highly correlated to perivascular fibroblast, pericyte, and SMC signatures but not to meningeal fibroblast signature (Figure 2G), likely reflecting their low frequency in GBM, which is consistent with rare cases of primary extracerebral meningeal GBM (Michaelson and Connerney 2020). Notably, the PV-FB cluster expressed several classical CAF markers such as *PDGFRA*, *PDGFRB*, *COL1A1*, and *FAP* (Sharon et al. 2013; Erez et al. 2010; Nurmik et al. 2020). Strikingly, we found that enrichment of perivascular fibroblasts was prognostic of poor clinical outcome in GBM (Figure S2G) and correlated with mesenchymal subtype (Figure S2F), suggesting their possible pro-tumorigenic role. Therefore, we refer to this population of cells as “CAF-like” whenever appropriate to reflect their CAF-like tumor-promoting phenotype.

Next, we applied SCENIC to infer active regulators of P-FB cells and confirm their identity in GBM. For PV-FB and M-FB cells, SCENIC predicted a high activity of several common brain fibroblast transcription factors ZIC1, FOXC1, NR2F2 (DeSisto et al. 2019), TWIST1 (García-Palmero et al. 2016), and a CAF-specific transcription factor NR2F1 (Figure 2F) (Wu et al. 2022). The activity of these regulons was restricted to cells with PV-FB and M-FB signatures, indicating that their phenotype is distinct from that of pericytes or smooth muscle cells (Figure 2E). These results suggest that fibrotic scarring in GBM may share some of its mechanisms with other CNS pathologies in which fibrogenic cells are derived from perivascular fibroblasts in response to inflammatory stimuli (David Oliveira Dias et al. 2018). Interestingly, however, our analysis of genomic copy number using the CopyKat algorithm identified a fraction of perivascular stromal cells as aneuploid (Figure S2D). Since mesenchymal differentiation of glioma stem cells (GSCs) into pericytes has been previously shown (deCarvalho et al. 2010; Ricci-Vitiani et al. 2010), these findings raise an intriguing possibility that malignant cells can undergo a mesenchymal differentiation to assume a CAF-like phenotype in GBM.

Next, to identify the presence of closely related stromal cell types in other ECM^hi^-enriched CNS tumors, we analyzed scRNA-seq data from neurofibroma (n=3), meningioma (n=7), low-grade pediatric tumors (LGPT; n=26), high-grade pediatric tumors (HGPT; n=23) and Bmets (n=15) (Figure 2I). Stromal/mesenchymal cells were detected at the highest frequency in BMets (21%), meningioma (26%), and neurofibroma (56%). We did not detect any stromal cells in HGPTs which our previous analysis identified to be ECM^hi^-enriched which could be ascribed to variability in cell isolation protocols, since detachment of cells embedded in the basement membrane requires stronger tissue dissociation methods (Vanlandewijck et al. 2018; Garcia et al. 2022).

In order to verify their fibrogenic phenotype, we applied SCIPAC on scRNA-seq and tumor-matched bulk RNA-seq data. SCIPAC predicted fibroblasts as the cellular source of fibrosis in neurofibroma and meningioma (Figure 2I). In Bmets, in addition to perivascular stromal cells, myeloid cells and endothelial cells were significantly associated with ECM^hi^ phenotype, suggesting their possible role in ECM remodeling. No ECM^hi^-associated cells were found in HGPT and LGPT tumors, consistent with the absence of stromal cell types in these datasets. Next, in order to identify conserved stromal cell states across different cancers, we performed the mutual nearest neighbors (MNN) batch correction on combined data and sub-clustered stromal cells based on MNN-corrected gene expression values (Figure S2I–J). The majority of GBM P-FB cells were clustered together with mesenchymal stem-like cells (MSCs) from Bmets. Indeed, perivascular CAF-like cells also upregulated mesenchymal progenitor markers *CTHRC1* and *ISLR* (Figure 2D) (Gonzalez et al. 2022), indicating that MSCs may be a source of CAF-like cells in GBM, as previously suggested (Jain et al. 2023). Neurofibroma fibroblasts, which have been characterized as distinct from classical fibroblasts (Brosseau et al. 2021), formed a separate cluster. Clusters 3, 4, and 5 predominantly contained meningioma mesenchymal subtypes together with meningeal GBM fibroblasts; cluster 0 consisted almost entirely of pericytes and smooth muscle cells. Overall, the results of this integrative analysis suggest that despite their common role in ECM remodeling of the tumor stroma, stromal cells exhibit distinct and largely non-overlapping phenotypes across different CNS malignancies.

Finally, to determine whether P-FB cells play a role in anti-tumor immunity and response to immunotherapy in GBM, we interrogated retrospective data from two recent anti-PD1 immunotherapy trials (Cloughesy et al. 2019; J. Zhao et al. 2019). In both cohorts, we found a significant reduction in overall survival for patients whose tumors were enriched in P-FB cells (Figure 2H), but not other mural cell subpopulations (Figure S2H), suggesting that the presence of P-FB cells is prognostic of a poor immunotherapeutic response.

### CAF-like cells activate chemoattractant signaling pathways to recruit tumor-associated macrophages to the TME

**Figure 3.**
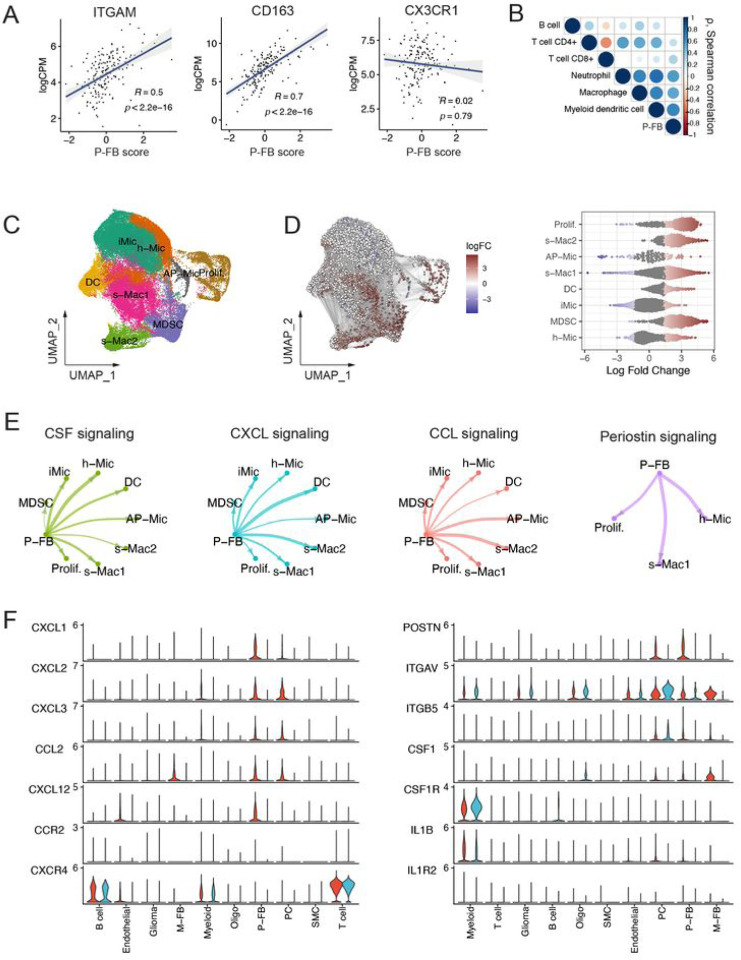
Perivascular fibroblasts secrete soluble factors that recruit myeloid cells from the circulation and polarize them toward an immunosuppressive phenotype. **(A)** Correlation between perivascular fibroblast (P-FB) signature score and monocyte-derived macrophage (ITGAM, CD163) and microglial (CX3CR1) markers. Value of the spearman correlation coefficient is shown together with the p value. **(B)** Spearman correlation between perivascular fibro- blast signature score and immune cells fractions in bulk RNA-seq data, estimated using the TIMER algorithm. **(C)** UMAP embedding of myeloid cells colored by cell subpopulation. **(D)** Differential abundance analysis of ECM^hi^ and ECM^lo^ tumors. The left panel shows a graph of cellular neighborhoods superimposed on the UMAP embedding of the data. Color represents log fold change (logFC) relative to ECM^lo^, node size represents the size of the neighborhood, and edge width represents the amount of overlap between neighborhoods. Relative changes which were not statistically significant (p > 0.05) are not shown. The right panel shows the distribution of differential abundance of cellular neighborhoods across myeloid subpopulations. **(E)** Interaction strength between P-FB cells and myeloid cells for chemoattractant pathways. Mac – macrophage; Mic – microglia; Prolif. – proliferating macrophage; MDSC – myeloid-derived suppressor cell; DC – dendritic cell. **(F)** Expression of selected ligands and their receptors for pathways from (E).

Next, we hypothesized that GBM P-FB cells may play a role in modulating neuroinflammation and contribute to the establishment of an immunosuppressive TME that is resistant to immune checkpoint blockade such as anti-PD1 antibodies. To verify this, we first performed cell type deconvolution of GBM bulk gene expression profiles. We found a strong positive correlation between the presence of P-FB cells and myeloid cells, including macrophages, dendritic cells, and monocytes (Figure 3A). P-FB cell signature was positively correlated to expression of CD11B (*ITGAM*) and *CD163*, but not *CX3CR1* (Figure 3B), indicative of increased numbers of monocyte-derived macrophages (Venteicher et al. 2017), as well as T cell exhaustion markers (Figure S3A). This was supported by differential abundance analysis of GBM scRNA-seq data, in which we found an enrichment of two populations of tumor-associated macrophages (TAMs) s-Mac1 and s-Mac2 (Figure S2B), myeloid-derived suppressor cells (MDSCs) and proliferating (Prolif.) macrophages in samples that were classified as ECM^hi^ based on pseudobulk expression profiles (Figures 3C, S2B, S3C). Additionally, macrophages, dendritic cells (DCs) and MDSCs displayed a lower expression of genes encoding MHC class II (MHC-II) molecules (Figure S3B), indicating their poor antigen-presenting capacity.

To investigate possible mechanisms of macrophage recruitment by P-FB cells, we applied the CellChat algorithm that can infer cell-state specific signaling communications from scRNA-seq data (Jin et al. 2021). CellChat predicted active chemoattractant signaling from P-FB cells to macrophages via chemokines CCL2, CXCL1, CXCL2, CXCL12, CSF1, and matricellular protein periostin (POSTN), which are known to induce chemotaxis and alternative polarization of tumor-supporting M2-like myeloid cells (Figure 3D–F) (Turley, Cremasco, and Astarita 2015; Sánchez-Martín et al. 2011; Shi et al. 2018; Zhou et al. 2015; Stafford et al. 2016; Flores-Toro et al. 2020). These results suggest that P-FB cells may contribute to the establishment of an immunosuppressive microenvironment by driving M2-like TAM recruitment and polarization via chemotaxis and periostin signaling, respectively.

### Perivascular fibroblasts promote immune-evasive stem-like cancer cell phenotype in GBM

**Figure 4.**
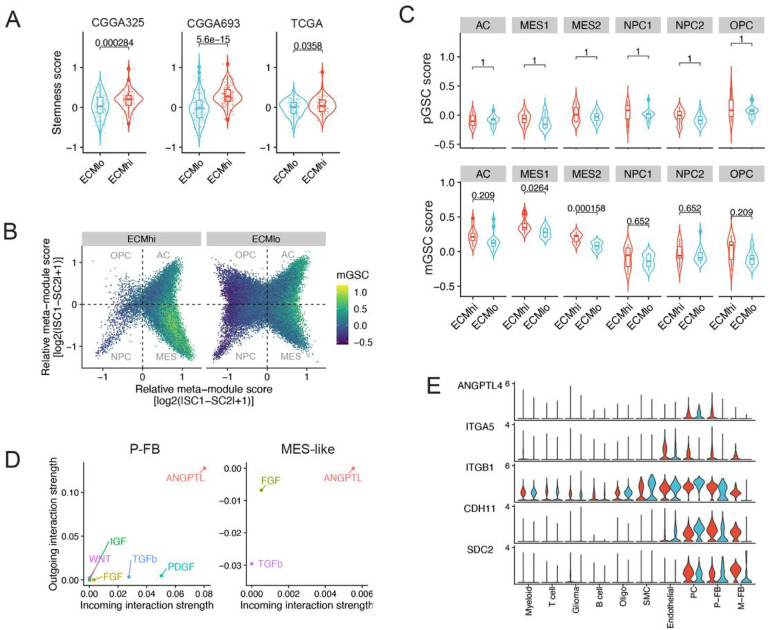
Perivascular fibroblasts secrete factors that upregulate stem-like programs in ECMhi glioblasto- ma. **(A)** Stemness signature scores in ECM^hi^ and ECM^lo^ tumors across three glioblastoma (GBM) RNA-seq datasets. **(B)** Two-dimensional butterfly plot of glioma cell states in ECM^hi^ and ECM^lo^ tumors, colored by enrichment of the mesenchymal glioma stem cell (mGSC) signature. OPC – oligodendrocyte progenitor cell-like; AC astrocyte-like; NPC – neural progenitor cell-like; MES – mesenchymal-like. **(C)** Proneural (top) and mesenchymal (bottom) glioma stem cell scores in ECM^hi^ and ECM^lo^ tumors. Two-sided t-test. Benjamini-Hochberg adjusted P values are shown. **(D)** Strength of outgoing and incoming signaling in perivascular fibroblasts (P-FB) and MES-like glioma cells for selected pathways with known role in cancer stem cell maintenance. **(E)** Expression of ANGPTL4 and its receptors in GBM scRNA-seq data.

GSCs are maintained within perivascular collagen-rich niches (Motegi et al. 2014; Calabrese et al. 2007), and are known to evade antitumor immune responses through various mechanisms, including downregulation of MHC class I, induction of quiescence, and other mechanisms that promote immune tolerance (McClellan et al. 2023). Therefore, we hypothesized that GBM P-FB cells can modulate anti-tumor immune responses through maintenance of GSCs in the perivascular niche. In order to verify this, we first quantified the stem cell-like tumor phenotype by computing “stemness score” (Miranda et al. 2019). We found that ECM^hi^ tumors displayed a higher stemness score across all three datasets analyzed (Figure 4A), suggesting that a fibrotic microenvironment may favor the emergence and/or maintenance of glioma stem-like cell phenotype. We then classified glioma cells into oligodendrocyte-progenitor-like (OPC-like), neural-progenitor-like (NPC-like), astrocyte-like (AC-like), and mesenchymal-like (MES-like) cell states (Neftel et al. 2019), and found that ECM^hi^ tumors were characterized by an enrichment of MES-like cells and reduced frequency of NPC-like and OPC-like states (Figure 4B, S3D), which is in line with the preponderance of myeloid cells in ECM^hi^ tumors and their proposed role in maintaining the MES-like cell state (Neftel et al. 2019).

GBM contains hierarchies of mesenchymal and proneural GSCs (mGSCs and pGSCs, respectively) that are considered largely responsible for cancer cell heterogeneity observed within GBM tumors (L. Wang et al. 2019). To understand the contribution of these progenitor states to glioma cell heterogeneity observed in ECM^hi^ and ECM^low^ subtypes, we scored single cells using previously identified mGSC and pGSC signature genes. We found that MES-like glioma cells in ECM^hi^ tumors were enriched in mGSCs (Figure 4B, **S4E**), which is in line with their increased frequency.

Next, to elucidate possible mechanisms of mGSC enrichment, we compared CellChat-inferred signaling networks between ECM^hi^ and ECM^lo^ tumors. We found an upregulation of ANGPTL, WNT, and FGF signaling from by P-FB cells to glioma cells, all of which have been implicated in maintenance of stem-like cell phenotype in GBM (Figure 4C–D) (Tsai et al. 2019; Lee et al. 2016; Muthukrishnan et al. 2022). MES-like glioma cells expressed multiple receptors for ANGPTL4, including syndecans SDC2, SDC3, and SDC4 as well as WNT5A receptor FZD6 (Figure 4E), suggesting that these pathways may be responsible for the enrichment of GSCs in ECM^hi^ tumors.

## Discussion

Previous studies have shown that therapeutically reducing ECM deposition can alleviate immunosuppression across various cancer models, including GBM (Datta et al. 2023; Y. Zhao et al. 2019; Chauhan et al. 2019). However, to date, comprehensive analyses of the ECM across different brain tumors, which could provide important insights into tumor progression and treatment resistance, have been limited. In this study, we undertake a pan-CNS analysis of the ECM transcriptome in brain tumors to identify which cellular and molecular components contribute to the formation of ECM stroma in CNS tumors. Our analysis identifies that brain tumor ECM can be described by a single axis of variation, ranging from highly fibrotic ECM^hi^ state to ECM-deplete ECM^low^ state. Brain tumors such as medulloblastoma and glioneuronal tumors are largely devoid of expression of ECM components, whereas neurofibroma and schwannoma were classified almost exclusively as ECM^hi^. Indeed, it is known that up to 50% of neurofibroma’s dry weight is collagen (Peltonen et al. 1986), which is consistent with our findings. Additionally, our findings in GBM are in line with a previous study that also identified a subset of GBM tumors characterized by overexpression of transcripts of ECM components and reduced patient survival (Freije et al. 2004).

Deposition of ECM components in GBM has been proposed to have three different origins: adjacent stromal cells, normal brain cells that are activated in response to tumor-derived factors (Knott et al. 1998), or by malignant cells as a part of their mesenchymal phenotype (Tso et al. 2006). In human GBM specimens, ECM-rich zones bordering cellular tumor regions and around blood vessels are characterized by high expression of smooth muscle actin (encoded by *ACTA2*) (Dinevska et al. 2022), suggesting the contribution of mesenchymal stromal cells to ECM deposition. Stromal cells with myofibroblast-like appearance and expression of CAF markers such as α-SMA and PDGFRβ have been found in histologically normal surgical margins and around blood vessels (Clavreul et al. 2012, 2014). Additionally, isolation of CAF-like cells from GBM tumor tissue has recently been reported (Galbo et al. 2022; Jain et al. 2023).

CAFs have been proposed to have diverse origins, including tissue-resident fibroblasts, bone marrow-derived MSCs, adipocytes, endothelial cells, and perivascular cells (P.-Y. Chen et al. 2021). In BMets, metastatic cells can bring their own soil – stromal components including activated fibroblasts – from the primary site to the brain (Duda et al. 2010). Until recently, CAFs were presumed to be absent in GBM, given the apparent paucity of fibroblasts in the CNS. However, recent efforts to comprehensively characterize human cerebrovasculature identified the presence of fibroblast-like cells in the human and murine brains (Vanlandewijck et al. 2018; Yang et al. 2022; Garcia et al. 2022; E. A. Winkler et al. 2022). In our present study, we identify two populations of fibroblasts in GBM that resemble previously identified brain perivascular and meningeal fibroblasts, suggesting that GBM CAFs likely arise from a local cellular source. While we cannot conclude that the cells identified in our analysis represent *bona fide* CAFs based solely on *in silico* results, P-FB cells express several CAF-specific gene markers (*PDGFRA*, *FAP*, *FN1*, *COL1A1*) and transcription factors (*NR2F1*) (Wu et al. 2022), which warrants further investigation of their identity and pro-tumorigenic roles in GBM.

Interestingly, we find that about 25% of P-FB cells carry the same genomic abnormalities as malignant glioma cells, indicating that at least some fraction of P-FB cells may have a neoplastic origin. GSCs are known to localize to perivascular niches and have the ability to undergo mesenchymal differentiation (deCarvalho et al. 2010; Ricci-Vitiani et al. 2010). Indeed, an analysis of human GBM specimens showed that GSCs can generate vascular pericytes upon stimulation with TGF-β, and that the majority of GBM pericytes are derived from malignant cells (Cheng et al. 2013). Whether GSCs can directly assume a fibroblast identity is still unknown; however, pericytes have been shown to undergo differentiation into stromal fibroblasts upon detachment of tumor microvasculature (Hosaka et al. 2016), as well as in kidney fibrosis (Y.-T. Chen et al. 2011). Therefore, it is possible to speculate that a fraction of GBM P-FB cells may originate from GSCs, either directly or through transdifferentiation from pericytes.

Although fibrotic scarring and inflammation by perivascular fibroblasts have been noted in traumatic brain injury (David O. Dias et al. 2021), they have been largely unappreciated as contributors to brain tumor stroma and antitumor immune responses. In the present study, we identify a perivascular origin of pathological fibroblasts in GBM and propose several mechanisms for fibroblast-mediated immunosuppression and immunotherapy resistance. To the best of our knowledge, our study is the first to report an association between the presence of CAF-like cells and immunotherapeutic outcomes in GBM. Here, we show that such CAF-like cells may simultaneously induce GSCs and reprogram the immune response to facilitate tumor immune evasion and immunotherapy resistance. GSCs are known to be less immunogenic, evade immune responses through the downregulation of MHC molecules and promote immune tolerance (McClellan et al. 2023). We also show that perivascular CAF-like cells can activate the production of chemoattractant molecules to recruit M2-like macrophages into the GBM TME, which promotes T cell exhaustion and immunosuppression. In addition to attraction and retention of tumor-promoting myeloid cells, CAFs are known to affect antitumor immune responses indirectly by production and remodeling of ECM components, which serves as a physical barrier restricting access of immune cells to cancer cells. Indeed, a recent immunohistochemical analysis of GBM tissue showed an enrichment of T cells in ECM-rich zones (Dinevska et al. 2022), suggesting that T cell trafficking to the tumor is impeded due to pathologically high deposition of ECM components by stromal cells in the perivascular niche. This has important implications for adoptive cell-based therapies, such as CAR-T and CAR-NK cell therapies. CAR-T cell therapy in GBM has shown limited success, partly due to limited CAR-T cells infiltration into the tumor when CAR-T cells are administered systemically (Mulazzani et al. 2019; O’Rourke et al. 2017). This suggests that overcoming these biophysical and biochemical barriers by targeting P-FB cells in GBM may help overcome immunotherapy resistance and increase patient survival.

## Methods

### scRNA-seq data processing and analysis

The scRNA-seq data (GSE182109) was obtained from Gene Expression Omnibus (Abdelfattah et al. 2022). Individual samples were log-normalized and integrated using Seurat’s reciprocal PCA (Hao et al. 2021). Doublets were identified and removed using Scrublet algorithm (Wolock, Lopez, and Klein 2019). Next, cells were clustered and annotated based on previously reported marker genes (Abdelfattah et al. 2022) as well as copy number alterations (CNAs) inferred using CopyKat algorithm (Gao et al. 2021). To exclude poor quality cells within each cell type, we applied median absolute deviation (MAD)-based outlier detection approach, as described previously (Germain, Sonrel, and Robinson 2020). Clusters characterized by low UMI counts, high fraction of mitochondrial reads, and uninformative marker genes were removed.

Malignant cells were assigned to meta-modules defined by Neftel et al. Briefly, single-cell scores were computed for each of the signatures (MES1-like, MES2-like, NPC1-like, NPC2-like, AC-like, OPC-like), and cells were assigned to each cell state, as previously described (Neftel et al. 2019).

### RNA-seq data processing and analysis

Publicly available RNA-seq data were downloaded from TCGA and CGGA databases. In total, three cohorts were used in this study: TCGA GBM, CGGA-693, and CGGA-325 (**Table 1**). The datasets were TMM-normalized, and logCPM values were used for all downstream analyses. The datasets were combined, and batch-corrected using *limma::removeBatchEffect* function.

Spatial distributions of ECM signatures were examined using the Ivy GAP dataset (Puchalski et al. 2018). Gene expression FPKM values were log transformed prior to analysis.

### Identification and validation of extracellular matrix subtypes

Batch-corrected logCPM values were mean-centered prior to clustering. Using the core matrisome geneset NABA_CORE_MATRISOME available in the Molecular Signature Database (MSigDB), we performed consensus non-negative matrix factorization (cNMF) with rank 2, as we observed the highest average cophenetic correlation coefficient for k=2. To validate the resulting clusters, we also performed consensus clustering using hierarchical clustering with average linkage (Monti et al. 2003), and observed high concordance between both algorithms. A set of 505 samples was recognized as core samples based on a positive silhouette coefficient.

In order to characterize ECM signatures and classify external samples, we generated subtype-specific gene signatures by searching for genes that were upregulated in all three datasets, according to the Wilcoxon test. As a result, a set of 49 signature genes for each subtype was obtained.

### Classification of external samples

External samples were classified into subtypes/signatures based on their signature scores, which were computed as previously described (Neftel et al. 2019). Since signature scores reflect up- or down-regulation of a gene signature compared to a control geneset, we classified samples into subtypes based on the sign of the signature score. For example, Samples scoring greater than 0 for gene signature of subtype A and lower than 0 for the signature of subtype B were classified as subtype A, samples scoring greater than 0 for gene signature of subtype B and lower than 0 for the signature of subtype A were classified as subtype B, and other samples are labeled as unidentified. For scRNA-seq data, pseudo bulk samples were generated, TMM-normalized, and classified as outlined above.

### Wang subtype classification

Uncorrected bulk RNA-seq data were classified into three molecular subtypes using the SubtypeME tool in the GlioVis portal (Q. Wang et al. 2017; Bowman et al. 2017). Each sample was assigned to a molecular subtype with the lowest p-value.

### Computation of cell-type-specific gene signatures

To estimate the enrichment of cell type-specific signature genes in bulk RNA-seq data, top marker genes were first identified for a cell type/state of interest using the FindMarkers function in the Seurat package (Hao et al. 2021). Genes expressed in fewer than 50% of cells and with fold change (FC) less than 2 were removed. Top 50 genes with the highest log2FC were considered as top marker genes. Bulk tumors were then scored for the resulting top marker genes as previously described (Neftel et al. 2019). Samples were classified as high or low for a cell type/state, if their signature score fell into the upper or lower quartiles, respectively. For the Cloughesy cohort, signature score of 1 was used as a cutoff.

## Supplementary Material

1
