# Supplementary material for: Pan-cancer transcriptomic analysis of CNS tumor stroma identifies a population of perivascular fibroblasts that predict poor immunotherapy response in glioblastoma patients
Source: Res Sq. 2023 May 15:rs.3.rs-2931886. Preprint. [Version 1] doi: 10.21203/rs.3.rs-2931886/v1 (PMC10246264; doi:10.21203/rs.3.rs-2931886/v1)
Supplement: 1 [file NIHPPRS2931886V1-supplement-1.pdf]

Figure S1. Identification and characterization of ECM subtypes in brain tumors. (A) First and second principal components of batch-corrected (left) and uncorrected (right) datasets. **(B)** Consensus matrix obtained from 50 runs of non-negative matrix factorization. **(C)** Consensus matrix obtained from hierarchical clustering. **(D)** Spearman correlation between gene expression clusters obtained using NMF and hierarchical clustering. **(E)** Metrics used for estimation of NMF factorization rank. **(F)** Principal component analysis of ECM^{hi}, ECM^{lo} and ECM^{int} brain tumor RNA-seq samples based on ECM gene expression. **(G)** Spearman correlation between median overall survival (OS) and average ECM^{hi} score in pediatric brain tumors. **(H)** Distribution of glioblastoma molecular subtypes in ECM^{hi} and ECM^{lo} tumors. **(I)** Signature gene expression in different anatomical regions of glioblastoma. **(J)** In situ hybridization and adjacent hematoxylin and eosin (H&E) sections annotated for different histologic regions of GBM tumors. ECM^{hi} hallmark genes *COL1A1*, *COL4A1* are expressed in CTmvp regions, suggesting that ECM^{hi} signature is spatially associated with GBM microvasculature. Labels on the left represent patient IDs. Anatomical regions are colored the same way as in (I).

Figure S2. Identification and characterization of perivascular fibroblasts in GBM scRNA-seq data. (A-C) Dot plots showing marker gene expression for different cell types. **(D)** Left: CopyKat predictions for all cell types; Right: CopyKat predictions for mural cells, grouped by ECM subtype, and shown both as frequency and absolute numbers. **(E)** UMAP embedding of GBM scRNA-seq data, colored by cell type. **(F)** Distribution of GBM molecular subtypes in tumors with high and low enrichment of the fibroblast-like signature. **(G)** Kaplan-Meier survival curves for ECM^{hi} (red) and ECM^{lo} (blue) GBM tumors. **(H)** Kaplan–Meier plot of overall and progression free survival for patients with high (red) or low (blue) presence of perivascular cell types (P values were calculated using log-rank test; PC – pericyte, SMC – smooth muscle cell, MFB – meningeal fibroblast). **(I)** UMAP embedding of batch-corrected stromal cell expression profiles from low-grade pediatric tumors, neurofibromas, meningiomas, and brain metastases, colored by cluster. **(J)** Barplot showing the per-cluster frequency of cell types from different tumors, related to (I). Red denotes MSC and fibroblast populations, blue denotes PC and SMC populations.

Figure S3. Immune signatures associated with perivascular fibroblasts. (A) Spearman correlation between the perivascular signature score and immune-related genes. Spearman correlation coefficients together with P value are shown for each pair. **(B)** Pro- and anti-inflammatory gene expression in different myeloid subpopulations, grouped by ECM subtype. The line divides anti-inflammatory (top) and pro-inflammatory (bottom) genes. **(C)** ECM^{hi} and ECM^{lo} signature scores of pseudobulk gene expression profiles, grouped by ECM subtype. **(D)** Frequency of glioma cell states in ECM^{hi} and ECM^{lo} tumors. OPC – oligodendrocyte progenitor cell-like; AC astrocyte-like; NPC – neural progenitor cell-like; MES – mesenchymal-like. Two-sided t-test. Benjamini-Hochberg adjusted P values are shown.
